# Supplementary material for: The Effect of the Ethanolic Extracts from Syzygium aromaticum and Syzygium nervosum on Antiproliferative Activity and Apoptosis in HCT116 and HT-29 Cells
Source: Int J Mol Sci. 2025 Jul 16;26(14):6826. doi: 10.3390/ijms26146826 (PMC12295921; doi:10.3390/ijms26146826)
Supplement: Supplementary file 1 [file ijms-26-06826-s001.zip › ijms-3679923-supplementary.pdf]

International Journal of *Molecular Sciences*

MDPI

Supplementary Materials

The Effect of the Ethanolic Extracts from Syzygium aromaticum and Syzygium nervosum on Antiproliferative Activity and Apoptosis in HCT116 and HT-29 Cells

Table S1: Flow Cytometry with Annexin V/PI staining in HCT116 and HT-29.

Extract compounds	Flow Cytometry with Annexin V/PI staining in HCT116			
	Live Cells (%)	Early Apoptosis (%)	Late Apoptosis (%)	Necrosis (%)
Control	87.06 ± 1.91	8.56 ± 2.41	1.06 ± 0.91	3.32 ± 0.99
SA 25 µg/ml	68.81± 1.99***	29.12 ± 1.84***	1.50 ± 0.09	0.58 ± 0.06
SA 50 µg/ml	69.08 ± 7.20***	29.04 ± 6.75***	1.12 ± 0.24	0.77 ± 0.22
SA 100 µg/ml	46.56 ± 1.11***	51.90 ± 6.75***	1.43 ± 0.27	0.13 ± 0.14
SA 200 µg/ml	49.66 ± 6.14***	46.00 ± 5.61***	2.88 ± 0.27	1.47 ± 0.44
SN 25 µg/ml	82.78 ± 2.09**	14.94 ± 2.50***	1.18 ± 0.23	1.10 ± 0.51
SN 50 µg/ml	76.01 ± 1.71***	22.93 ± 1.50***	0.88 ± 0.43	0.18 ± 0.04
SN 100 µg/ml	70.66 ± 0.64***	27.71± 0.75***	1.29 ± 0.59	0.35 ± 0.07
SN 200 µg/ml	64.99 ± 2.31***	31.69 ± 2.82***	2.10 ± 0.13	1.21 ± 0.76
	Flow Cytometry with Annexin V/PI staining in HT-29			
Control	96.21 ± 0.32	1.87 ± 0.06	0.73 ± 0.22	1.18 ± 0.16
SA 25 µg/ml	96.16 ± 0.56	1.71 ± 0.28	0.61 ± 0.14	1.52 ± 0.27
SA 50 µg/ml	95.43 ± 0.86	1.68 ± 0.27	0.29 ± 0.11	2.60 ± 0.63
SA 100 µg/ml	81.19 ± 0.76***	14.59 ± 0.61***	1.61 ± 0.25	2.61 ± 0.52
SA 200 µg/ml	73.42 ± 2.17***	22.12 ± 1.10***	1.88 ± 0.29	2.58 ± 0.92
SN 25 µg/ml	91.62 ± 0.25	1.87 ± 0.06**	0.73 ± 0.22	1.18 ± 0.16
SN 50 µg/ml	83.47 ± 1.02***	3.25 ± 0.24***	1.10 ± 0.08	4.03 ± 0.42
SN 100 µg/ml	81.15 ± 0.63***	8.41 ± 0.44***	3.15 ± 0.73	4.97 ± 0.43
SN 200 µg/ml	74.08 ± 1.03***	14.72 ± 0.56***	1.54 ± 0.24	2.58 ± 0.42

Data are presented as mean ± SD (n = 3). Statistical analysis was performed using one-way ANOVA, followed by a post hoc test comparing each treatment to the control group. *** $p < 0.001$.

Table S2. Fluorescence intensity of Caspase-3/7 activity.

Extract compounds	Caspase 3/7 Fluorescence Intensity				
	**Control**	**25 μg/ml**	**50 μg/ml**	**100 μg/ml**	**200 μg/ml**
			**HCT116**		
***Syzygium aromaticum* (SA)**	283.75 ± 132.81	1660 ± 450.60	4019.38 ± 141.70	21473 ± 8984.16**	41660 ± 4256.39***
***Syzygium nervosum* (SN)**	283.75 ± 132.81	813.02 ± 251.06	4687.44 ± 1012.29**	6494.22± 2262.68***	7431.19 ± 610.16***
			**HT-29**		
***Syzygium aromaticum* (SA)**	226.53 ± 41.07	1594.93 ± 492.12	9600 ± 2589.94*	36730.07 ± 4571.27***	25780.27 ± 4745.31***
***Syzygium nervosum* (SN)**	226.53 ± 41.07	2536.70 ± 771.69	3185.99 ± 839.80*	8013.38 ± 333.25***	18939.43 ± 1134.74***

Data are presented as mean ± SD (n = 3). Statistical analysis was performed using one-way ANOVA, followed by a post hoc test comparing each treatment to the control group. ** $p < 0.01$, and *** $p < 0.001$.
